# Going commando as part of a multifaceted intervention to reduce CAUTIs in critically ill children

**DOI:** 10.1017/ash.2024.456

**Published:** 2025-01-06

**Authors:** Matthew Linam, Lisette Wannemacher, Kathryn Powell, Christina Calamaro, Karen Walson

**Affiliations:** 1 Division of Pediatric Infectious Diseases, Emory University School of Medicine, Atlanta, GA, USA; 2 Children’s Healthcare of Atlanta, Atlanta, GA, USA; 3 Nell Hodgson Woodruff School of Nursing, Emory University, Atlanta, GA, USA

## Abstract

This project was initiated in a large pediatric intensive care unit to reduce catheter-associated urinary tract infections (CAUTIs). Implementing removal of diapers and a urine collection device that prevented urine backflow in March 2021 decreased the rate from 3.3 to 0.9 CAUTIs/1000 catheter-days. These interventions could augment CAUTI prevention strategies.

Catheter-associated urinary tract infections (CAUTIs) are a source of preventable harm in children resulting in prolonged hospitalization and excess healthcare costs.^1^ Insertion and maintenance bundles have significantly reduced CAUTIs, but infections still occur.^2,3^ In a cross-sectional study from 2013 to 2018 reporting population-based CAUTI rates in children, there was a statistically significant gradual decrease in CAUTI rates and catheter days.^4^ However, National Healthcare Safety Network (NHSN) data from during the coronavirus disease 2019 pandemic showed increased CAUTIs.^5^ We experienced an increase in CAUTIs from a historic baseline rate of 0.5/1000 catheter-days to 3.3/1000 catheter-days from July 2019 through February 2021. The objective of this project was to identify preventable causes of CAUTI and develop and test interventions to reduce them.

## Methods

This quality improvement project was initiated in the 56-bed PICU of a 370-bed tertiary academic children’s hospital that is part of a large pediatric health system in the southeastern US. Since 2013, CAUTI prevention has been a system-wide safety focus. In 2013, the healthcare system implemented evidence-based prevention bundles for CAUTI (catheter insertion and maintenance bundles).^2,6^ In 2017, the hospital implemented a hygiene bundle (oral care based on patient needs, daily bath, linen change, and cleaning of high-touch surfaces) primarily for the prevention of central line-associated bloodstream infections (CLABSIs); although, some components were applicable to CAUTI prevention. Starting in October 2019, interdisciplinary infection prevention rounds led by the hospital epidemiologist and unit nursing leader with the bedside nurse, occurred weekly for patients with urinary catheters in place for greater than three days. Internal infection data suggested that the risk of CAUTI increased when a urinary catheter had been in place for greater than three days. The framework for infection prevention rounds had previously been implemented to prevent CLABSIs in critically ill children and this process has been previously reported.^7^ Discussions included strategies to optimize maintenance of the urinary catheter and identify catheters that could be removed.

As part of ongoing improvement efforts, all CAUTIs on the unit are reviewed by a multi-disciplinary team comprised of infection prevention, the hospital epidemiologist, the unit nurse quality leader, other nursing staff and the physician medical director. In most infections, opportunities for more consistent prevention bundle compliance or earlier catheter removal were noted. With the increase in CAUTIs that occurred between July 2019 and February 2021, an improvement team was formed that included the nurse quality leader, hospital epidemiologist, and physician medical director. The improvement team discussed practice differences with the other PICU in our healthcare system, which had not seen an increase in CAUTIs during that same time frame. The main difference identified was that the other PICU did not use diapers in children with urinary catheters. Interventions were developed and tested using the Plan-Do-Study-Act framework. In March 2021, children in the PICU with a urinary catheter were not diapered but were instead placed on an absorbent pad. Starting in August 2021, we began using a urine collection device that prevented both urine stasis in the drainage tube and retrograde flow of urine into the bladder (Accuryn™) on all children with a urinary catheter in place for three or more days. The device connected to the drainage catheter and primarily facilitated accurate measurement of urine output, but it had an added benefit of preventing urine backflow. Education related to these practice changes was sent out to the nursing staff and the practices were reinforced during interdisciplinary rounds.

CAUTI prevention bundle compliance was measured by direct observation by the nursing staff. Healthcare worker hand hygiene was recorded by covert direct observation by trained healthcare worker volunteers and recorded real-time using an electronic tool. CAUTIs were identified by prospective surveillance by infection prevention using standard NHSN definitions. Monthly catheter days were shown on a run chat. The CAUTI rate (infections per 1000 catheter-days) was reported monthly using a statistical process control chart. January 2017 through June 2019 served as the historical baseline. July 2019 through February 2021 was the period of increased CAUTIs, and March 2021 through February 2024 was the intervention period. Changes in catheter days and CAUTI rate over time that were statistically unlikely to have occurred by chance were identified using standard rules.^8^

The Children’s Healthcare of Atlanta Institutional Review Board reviewed and approved this project as non-human patients’ research.

## Results

**Figure 1.**
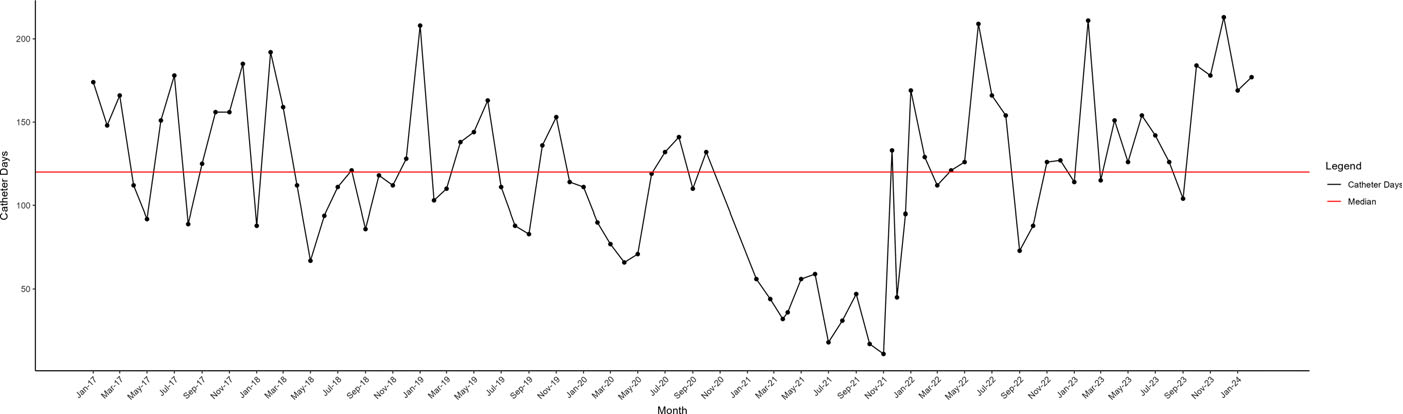
Run Chart Showing Urinary Catheter Days by Month in the Pediatric Intensive Care Unit from January 2017 through February 2024.

**Figure 2.**
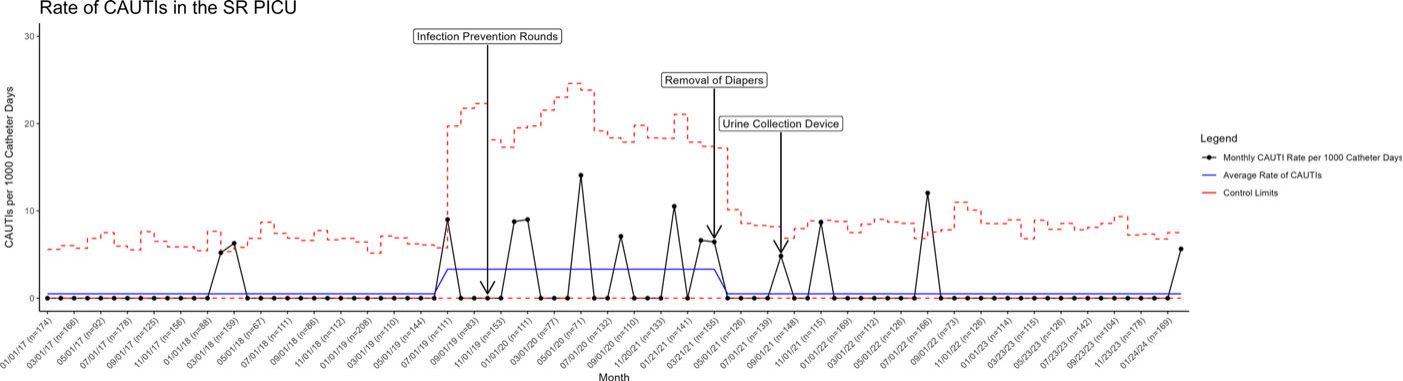
Statistical Process Control Chart Showing the Catheter-Associated Urinary Tract Infection Rate by Month in the Pediatric Intensive Care Unit from January 2017 through February 2024.

The baseline CAUTI rate (January 2017 – June 2019) was 0.5 infections/1000 catheter-days with an average of 349 days between CAUTIs. Between July 2019 and February 2021, the CAUTI rate increased to 3.3/1000 catheter-days with an average of 88 days between CAUTIs. During each of the three periods, the annual compliance with hand hygiene and the CAUTI prevention bundle elements remained above 90%. There was a special cause decrease in catheter days in 2020 and 2021 and a trend toward increased catheter days in 2023 (Figure 1). Starting in March 2021 after removal of diapers and implementation of the urine collection device that prevented retrograde flow, the CAUTI rate decreased to 0.9/1000 catheter-days and an average of 200 days between CAUTIs. The maximum days between CAUTIs was 573 days (Figure 2).

## Discussion

In this project, we demonstrated a 73% reduction in CAUTIs after removing diapers in children with urinary catheters and use of a urine collection device that prevented retrograde flow of urine into the bladder. In addition, after implementation of the interventions the unit went over 1.5 years without a CAUTI.

Although there was no decrease in CAUTIs that occurred after CAUTI prevention was included in interdisciplinary rounds, it is likely that rounds facilitated the implementation of the two project interventions. Discussion during rounds also led to earlier removal of some catheters and may have reinforced basic CAUTI prevention practices. The use of the urine collection device prevented inadvertent urine backflow that could occur during routine care.

The removal of diapers for children with a urinary catheter may seem counterintuitive at first as the lack of a diaper could lead to greater stool contamination, but we think that this had the opposite effect. The lack of a diaper likely led to earlier recognition of a bowel movement and prompt cleaning of the perineal area and urinary catheter. In addition, even when left unfastened, diapers would direct stool forward toward the catheter increasing the potential for enteric pathogen contamination. As previously noted, the other PICU in our system had already adopted this practice, and they were maintaining an average CAUTI rate of 0.7 in 2020 and 2021. Removal of diapers in children with urinary catheters resulted in a 75% decrease in CAUTIs at another children’s hospital, but to our knowledge, there are no other reports utilizing this practice.^9^ Current CAUTI prevention guides recommend routine hygiene, but there are no standard recommendations regarding diapers in patients with indwelling catheters.^2,3^

There were a couple of limitations with this project. This project was implemented in the PICU of a single children’s hospital and regional and cultural differences of other healthcare institutions may affect the success of the interventions. Although no other CAUTI interventions occurred during the intervention period of the project, factors such as additional nursing education or training may have affected outcomes. Despite decreased CAUTIs occurring after implementation of the two interventions, the rate did not decrease to the historical baseline. This was likely due to an increase in new inexperienced nurses in the PICU that began during the pandemic and continued through the intervention period.

In addition to currently recommended prevention practices, avoiding diapers in patients with indwelling urinary catheters and utilizing urine collection systems that prevent urine backflow could further reduce CAUTIs. Removal of diapers likely reduced stool contamination around the catheter and urethral opening. Further study is needed to determine whether these interventions should be considered standard practice.
